# Early administration of ivabradine in patients admitted for acute decompensated heart failure

**DOI:** 10.3389/fcvm.2022.1036418

**Published:** 2022-11-29

**Authors:** Teng-Yao Yang, Meng-shu Tsai, Jeng-Yu Jan, Jung-Jung Chang, Chang-Ming Chung, Ming-Shyan Lin, Hui-Ming Chen, Yu-Sheng Lin

**Affiliations:** ^1^Division of Cardiology, Department of Internal Medicine, Chang Gung Memorial Hospital, Chiayi, Taiwan; ^2^Department of Internal Medicine, Chang Gung Memorial Hospital, Chiayi, Taiwan; ^3^Center for Big Data Analytics and Statistics, Chang Gung Memorial Hospital, Linkou Medical Center, Taoyuan, Taiwan

**Keywords:** heart failure, ivabradine, atrial fibrillation, heart rate, acute heart failure

## Abstract

**Background:**

Heart rate (HR) control is important in heart failure (HF) patients with reduced ejection fraction, and ivabradine is indicated for patients with chronic HF and sinus rhythm. However, ivabradine is limited in initiation of ivabradine at acute stage of HF.

**Materials and methods:**

This multi-institutional retrospective study enrolled 30,639 patients who were admitted for HF from January 01, 2013 to December 31, 2018 at Chang Gung Memorial Hospitals. After applying selection criteria, the eligible patients were divided into ivabradine and non-ivabradine groups according to the initiation of ivabradine at the index hospitalization. HR, clinical outcomes including HF hospitalization, all-cause hospitalization, mortality, the composite of cardiovascular (CV) death or HF hospitalization and newly developed atrial fibrillation, and left ventricular ejection fraction (LVEF) and left atrium size were compared between the ivabradine and non-ivabradine groups after inverse probability of treatment weighting (IPTW) analysis after 12 months.

**Results:**

The HR at admission in the ivabradine group (*n* = 433) was 99.04 ± 20.69/min, compared to 86.99 ± 20.34/min in the non-ivabradine group (*n* = 9,601). After IPTW, HR was lower in the ivabradine group than that in the non-ivabradine group after 12 months (74.14 ± 8.53 vs. 81.23 ± 16.79 bpm, *p* = 0.079). However, there were no significant differences in HF hospitalization (HR = 1.02; 95% CI, 0.38–2.79), all-cause hospitalization (HR = 0.95; 95% CI, 0.54–1.68), mortality (HR = 0.87; 95% CI, 0.69–1.08), the composite of CV death or HF hospitalization (HR = 0.87; 95% CI, 0.69–1.08) and newly developed AF between the two groups. In addition, LVEF increased with time in both groups, but there were no significant differences during the observation period.

**Conclusion:**

Ivabradine was beneficial in controlling HR when initiated in patients with acute stage of HF, but it did not seem to provide any benefits in reducing HF hospitalization, all-cause hospitalization, and mortality in 1 year after discharge.

## Introduction

Heart failure (HF) with reduced left ventricular ejection fraction accounts for approximately half of HF patients (1). Heart rate (HR) is a significant prognostic factor for these patients with sinus rhythm (2–4), and β-blockers are essential medications. However, the percentage of patients who can tolerate an optimal dose of β-blockers is low due to complications such as compromised hemodynamics (5). In acute decompensated HF, tachycardia usually appears to compensate for decreased cardiac output, however, an increased HR is usually a serious condition in patients with decompensated HF (6). Hence, HR control is an important management goal in patients with decompensated HF. However, β-blockers impair cardiac inotropy and further worsen symptoms associated with acute decompensated HF, and are thus relatively contraindicated for patients with acute decompensated HF (7).

Ivabradine is a specific inhibitor of I*_*f*_* current in the sinoatrial node, and it can enhance left ventricular (LV) stroke volume and reverse LV remodeling in chronic HF simply by reducing HR (8, 9). The large “Systolic Heart failure treatment with the I*_*f*_* inhibitor ivabradine” (SHIFT) trial demonstrated the benefits of ivabradine for patients with chronic HF with a HR > 70 beats per minute (bpm) in combination with standard therapy including β-blockers, angiotensin converting enzyme inhibitors (ACEis), angiotensin receptor blockers (ARBs), and mineralocorticoid receptor antagonists (MRAs) (10). Compared with β-blockers, ivabradine does not impair cardiac contractility and does not affect hemodynamics (11, 12). A few studies have investigated the effect of ivabradine in the early phase of acute decompensated HF, and they have reported that the early co-administration of ivabradine during hospitalization for acute decompensated HF was well-tolerated and significantly reduced HR (5, 13, 14) even under dobutamine treatment (15, 16). However, these studies have mostly focused on HR and safety issues without reporting clinical outcomes. Therefore, we conducted this study to evaluate the effects of initiating ivabradine during the index hospital admission on clinical outcomes in patients with acute decompensated HF.

## Materials and methods

### Study population

This retrospective, multi-institutional, cohort study selected patients who were first admitted for HF from January 01, 2013 to December 31, 2018 in Chang Gung Medical hospitals, including four tertiary care medical centers and three major teaching hospitals around Taiwan with approximately 280,000 patients admitted per year and a total of 10,050 beds (17). Data on diagnoses, laboratory results, medications, echocardiography, and detailed chart records of each patient were collected from the CGMH medical database including medical records in hospitalization and out-patient visits, and which has been described in detail elsewhere (18, 19). The study protocol was approved by the Institutional Review Board of CGMH (IRB: 201900572B0C602).

**FIGURE 1:**
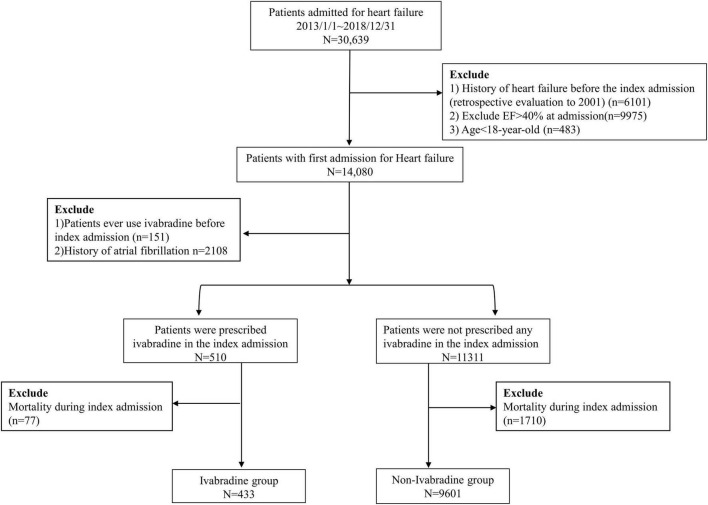
Flowchart of the study design.

A total of 30,639 patients with no previous diagnosis of HF at clinic visits before the index admission were enrolled in this study (a history of HF could be traced back to January 01, 2001). The exclusion criteria were: (1) left ventricular ejection fraction (LVEF) > 40% as the selected criteria of SHIFT-type patients in Swedish HF registry (20) or missing echocardiographic data according to echocardiography at the index admission (5, 21); and (2) age < 18 years. After applying these criteria, 14,080 patients remained. The European Society of Cardiology guidelines and enrollment criteria of the SHIFT study (10, 22) recommend that ivabradine should be prescribed in patients in sinus rhythm. Therefore, we further excluded patients with a previous diagnosis of atrial fibrillation (AF). To investigate the efficacy of initiating ivabradine in the acute stage of HF, we excluded patients who had ever used ivabradine before the index admission. The remaining 11,821 patients were included in this study, of whom 510 were prescribed with ivabradine at the index admission (ivabradine group) and 11,311 patients were not (non-ivabradine group). We then excluded the patients who died during the index admission because the aim of our study focused on outcomes after discharge. Finally, 10,034 patients were eligible for the study, of whom 433 were prescribed with ivabradine and 9,601 were not during the index admission. The study flowchart is shown in Figure 1.

### Assessment and definitions of outcomes

The index admission was defined as patients who were first admitted for heart failure with ejection fraction ≦ 40% from January 01, 2013 to December 31, 2018, and the following outcomes were assessed during a 12-month observation period after discharge. The primary endpoint was HF hospitalization. Other clinical outcomes included all-cause hospitalization, mortality, the composite of cardiovascular (CV) death or HF-hospitalization and newly developed AF. Although we enrolled the patients admitted to our hospitals during January 01, 2013 to December 31, 2018 but the medical records can be traced back to January 01, 2001. Therefore, the newly developed AF was defined as the diagnosis of AF was recorded after the index day and it never was recorded before the index day. In addition, the diagnosis of AF was confirmed by any medical records, such as report of electrocardiography, echocardiogram and 24-h electrocardiography. The definition of heart failure hospitalization based on the medical records and principal diagnosis for admission. All-cause hospitalization was defined as any admission after index day. The definition of CV death was the criteria of the Standardized Definitions for Cardiovascular and Stroke Endpoint Events in Clinical Trials by the FDA in the United States (23).

In addition, LV systolic function (LVEF), LV end-diastolic dimension, and left atrium (LA) size were also assessed. HF hospitalization was defined as an unscheduled hospitalization with new or worsening symptoms or signs, and diagnostic testing results consistent with the diagnosis of HF. In addition, a significant change in the treatment of HF, defined as a significant change in oral diuretics, the initiation of intravenous diuretics or intravenous vasoactive agents, or receiving mechanical ventilation or mechanical support was also required to define HF hospitalization (10, 23).

Data of HR were also extracted at the 1st, 3rd, 6th, and 12th months of follow up in outpatient clinics. HR at the index discharge was also assessed, as well as the change in HR, which was defined as the HR at the index discharge minus the HR at the index admission. The echocardiographic results, including LVEF, left ventricular end diastolic dimension (LVEDD), and LA size, were assessed from parasternal or apical views using the standard M-mode or 2D Simpson method in transthoracic echocardiography.

### Covariates

The covariates were vital signs (HR and blood pressure), demographics (age and sex), etiology of HF, comorbidities (including hypertension, diabetes mellitus, dyslipidemia, gout, chronic obstructive pulmonary disease and end-stage of renal disease, and previous hospitalization for myocardial infarction or stroke), baseline echocardiography (LVEF, LVEDD, and LA size), baseline laboratory data (including hemoglobin and creatinine), and the use of medications (including β-blockers and diuretics and so on). A complete list of the covariates is shown in Table 1. The Comorbidities were defined according to discharge diagnosis and/or two outpatient visits. The baseline laboratory data was retrieved from the initial blood testing in the index admission, while baseline echocardiography included echocardiography performed within 1 month before index admission or in index admission. The information of these covariates was extracted from outpatient and inpatient claims data (for diagnosis), laboratory records, echocardiography, pharmacy records, and detailed chart records from the CGMH medical databases.

**TABLE 1:** Baseline characteristics of the patients with heart failure with reduced ejection fraction.

	Before propensity score weighting			After propensity score weighting^†^		
Variables	Ivabradine group (*n* = 433)	Non-ivabradine group* (*n* = 9,601)	SMD	Ivabradine group (*n* = 433)	Non-ivabradine group* (*n* = 9,601)	SMD
Age (years)	62.09 ± 16.92	68.36 ± 15.55	0.386	67.94 ± 13.41	68.12 ± 15.63	0.012
Sex (Male), %	71.36	60.06	0.24	63.4	60.51	0.06
Etiology of heart failure						
Ischemic heart disease	61.89	50.8	0.225	54.66	51.19	0.07
Non-ischemic cardiomyopathy	38.11	49.2	0.225	45.34	48.81	0.07
Comorbidity, %						
Hypertension	56.35	68.81	0.26	67.97	68.36	0.008
Diabetes mellitus	16.63	32.40	0.373	32.32	31.82	0.011
Dyslipidemia	30.95	29.22	0.038	33.58	29.26	0.093
Gout	9.24	11.85	0.085	11.17	11.77	0.019
COPD	12.70	14.52	0.053	13.39	14.4	0.029
Dialysis	12.70	17.95	0.146	16	17.71	0.046
History of event						
Ischemic stroke	10.85	15.01	0.124	9.76	14.87	0.156
Myocardial infarction	36.72	22.92	0.305	27.88	23.4	0.103
Heart rate at admission	99.04 ± 20.69	86.99 ± 20.34	0.587	97.43 ± 17.46	87.23 ± 20.46	0.536
Echocardiography						
LVEF (%)	27.33 ± 7.93	30.86 ± 7.57	0.455	30.21 ± 4.51	30.58 ± 7.82	0.058
LA size (mm)	42.44 ± 7.75	42.57 ± 9.95	0.015	42.08 ± 6.51	42.62 ± 9.89	0.064
LVEDD (mm)	55.85 ± 16.47	57.49 ± 10.55	0.119	53.93 ± 10.81	57.62 ± 10.89	0.340
Laboratory data at admission						
HbA1C (%)	6.83 ± 1.52	6.82 ± 1.63	0.006	6.76 ± 1.07	6.84 ± 1.68	0.057
Hemoglobin (g/dl)	12.71 ± 2.53	11.97 ± 2.62	0.287	12.26 ± 1.98	12.02 ± 2.65	0.103
Creatinine (mg/dl)^‡^	1.11	1.14	0.096	1.22	1.13	0.000
eGFR^‡^	65.86	64.51	0.06	58.04	64.74	0.143
ALT (U/L)^‡^	32.00	26.00	0.143	31.00	26.00	0.145
BNP (pg/mL)^‡^	1370.00	1139.00	0.088	1860.00	1140.00	0.278
Medication at discharge, %						
ACEi or ARB	77.83	60.47	0.037	61.89	61.13	0.016
Non-dihydropyridine CCB	7.39	10.91	0.227	10.35	10.88	0.017
Dihydropyridine CCB	24.02	31.83	0.136	20.21	31.54	0.261
ß-blocker	89.15	71.07	0.181	75.59	71.75	0.087
Digoxin	17.09	15.68	0.184	20.19	15.95	0.11
Thiazide	5.54	5.69	0.113	3.24	5.68	0.118
Loop diuretic	94.69	71.92	0.221	90.91	72.4	0.493
MRA	67.67	34.07	0.152	55.15	34.8	0.418
Sacubitril/Valsartan	15.01	2.77	0.071	10.87	2.9	0.319
Statin	51.96	39.50	0.09	39.96	39.93	0.001
DPP4i	24.48	21.39	0.091	23.31	21.37	0.047
Biguanides	24.71	18.84	0.046	16.48	19.03	0.067
Sulfonylurea	4.39	10.13	0.182	6.15	10.04	0.143
SGLT2i	7.39	1.90	0.038	2.06	2.1	0.003
Insulin	40.65	30.97	0.101	46.91	30.91	0.333
Anti-platelet agent	76.67	63.37	0.002	76.33	63.78	0.277
Admission stay (days)	15.86 ± 13.75	11.62 ± 10.88	0.342	15.01 ± 11.48	11.67 ± 10.89	0.299
ICU stay (days)	8.17 ± 6.67	6.58 ± 6.14	0.248	11.38 ± 10.88	6.59 ± 6.15	0.542

ACEi, angiotensin converting enzyme inhibitor; ALT, alanine aminotransferase; ARB, angiotensin II receptor blocker; BNP, B-type natriuretic peptide; CCB, calcium channel blocker; COPD, chronic obstructive pulmonary disease; DPP4i, dipeptidyl peptidase-4 inhibitor; LVEF, left ventricular ejection fraction; HbA1C, hemoglobin A1c; ICU, intensive care unit; LA, left atrium; LVEDD, left ventricular end-diastolic dimension; MRA, mineralocorticoid receptor antagonist; SGLT2i, sodium-glucose cotransporter 2 inhibitor; SMD, standardized mean difference.

*Ivabradine group: Patients treated with ivabradine during the index hospitalization; Non-ivabradine group: Patients not treated with ivabradine during the index hospitalization. Categorical data are presented as %; Continuous data are expressed as mean ± standard deviation.

^†^Used age, hypertension, diabetes mellitus, ischemic heart disease, dyslipidemia, gout, COPD, dialysis, history of event-myocardial infarction, HbA1C, hemoglobin, creatinine, EF, BNP, statins, beta-blockers, biguanides, admission stay, and ICU stay to calculate the propensity score.

^‡^Those variables were expressed as median.

### Statistics

We created an inverse probability of treatment weighting (IPTW)-adjusted cohort based on propensity score to achieve comparability between the study groups (ivabradine vs. non-ivabradine) when comparing outcomes. The propensity score was calculated using multivariable logistic regression where the study group was regressed on most of the covariates listed in Table 1. We used a stabilized weight to mitigate the impact of extreme propensity scores (24). The balance of covariate distribution between groups was checked using the absolute value of the standardized difference (STD) (25) before and after weighting, where a value of < 0.2 was considered to be a small difference. In addition, due to the existence of missing echocardiography and laboratory data, the missing values were first imputed using the single expectation–maximization (EM) imputation method, and IPTW was conducted using the imputed data. The cumulative incidence of heart failure hospitalization, all-cause hospitalization, and the newly developed AF among the study groups was compared using the Fine and Gray subdistribution hazard model, which considers mortality during follow up as a competing risk.

The data of echocardiography (LVEF, LVEDD, and LA) at 12 months and HR at follow-up visits between groups were compared using a linear regression model. A two-sided *P*-value of < 0.05 was considered to be statistically significant. All statistical analyses were performed using SAS Version 9.4 (SAS Institute, Cary, NC, USA).

## Results

### Patient characteristics

Of the 10,034 patients who were eligible for the study, 433 patients initiated ivabradine during the index admission (ivabradine group) and 9,601 patients did not (non-ivabradine group). Table 1 shows the baseline characteristics of the two groups. The ivabradine group were significantly younger and had a higher percentage of males compared to the non-ivabradine group. The mean HR in the ivabradine group was 99.04 ± 20.69 bpm at admission, which was significantly higher than that in the non-ivabradine group (86.99 ± 20.34 bpm; STD = 0.587). The ivabradine group also had lower rates of comorbidities including diabetes mellitus and hypertension. In addition, the ivabradine group had worse LV function compared to the non-ivabradine group (LVEF 27.33 ± 7.93% vs. 30.86 ± 7.57%; STD = 0.455). In terms of medications, the ivabradine group had a significantly higher prescription rates of loop diuretics compared to the non-ivabradine group (94.69 vs. 71.92%; SMD = 0.221). The prescription rates of ACEis/ARBs, β-blockers, digoxin, aldosterone antagonists, and sodium glucose cotransporter 2 inhibitors (SGLT2is) were also higher in the ivabradine group, although the differences were not significant. In addition, the ivabradine group had a significantly longer hospital stay (15.86 ± 13.75 vs. 11.62 ± 10.88 days; STD = 0.342) and intensive care unit stay (8.17 ± 6.67 vs. 6.58 ± 6.14 days; STD = 0.248) compared with the non-ivabradine group. After IPTW adjustment, most covariates listed in Table 1 were well-balanced between the two groups.

### Heart rate and blood pressure during the observation period

**FIGURE 2:**
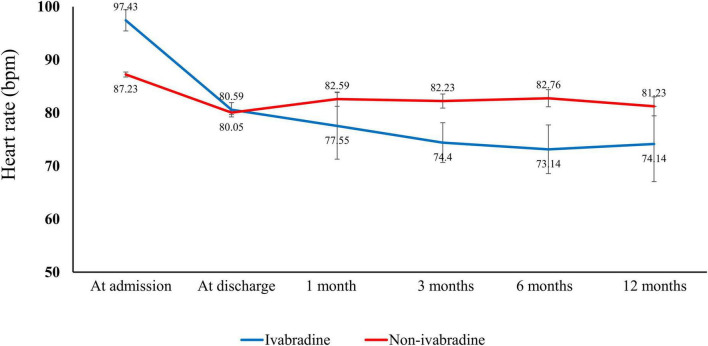
Heart rate in the index admission and observation period. There was a higher heart rate in the ivabradine group than in the non-ivabradine group at admission. However, the heart rate was lower in the ivabradine group after discharge.

The HR at admission, discharge, and at 1, 3, 6, and 12 months in both study groups are summarized in Figure 2 and Supplementary Table 1. Although there was a significantly higher HR at admission in the ivabradine group, there was no significant difference at discharge compared with the non-ivabradine group (80.59 ± 11.78 vs. 80.05 ± 15.12 bpm; *P* = 0.443). The decrease in HR was significantly greater in the ivabradine group during hospitalization than in the non-ivabradine group (*P* < 0.0001). After discharge, the mean HR was significantly lower in the ivabradine group than in the non-ivabradine group at 1, 3, 6, and 12 months. In terms of dosage of ivabradine, the adjustment of dosing was according to the target of heart rate. There were 61% of patients in ivabradine group continued to take ivabradine with the average dose of 8 mg per day 1 month after discharge, and there were 35% of patients continued to take ivabradine with the mean dose of 8 mg 3 months after discharge.

During the study period, the systolic arterial blood pressure was lower in ivabradine group than that in non-ivabradine group at discharge [120.98 ± 19 mmHg in ivabradine group vs. 128.54 ± 32.08 mmHg in non-ivabradine group (*P* < 0.001)] and 1 month after discharge [117.37 ± 18.85 mmHg in ivabradine group vs. 128.11 ± 26.26 mmHg (*P* < 0.001)]. However, there were no significant differences at 12-month follow-up [133.54 ± 18.49 mmHg in ivabradine group vs. 130.87 ± 28.53 mmHg in non-ivabradine group (*P* = 0.265)].

### Clinical outcomes

**FIGURE 3:**
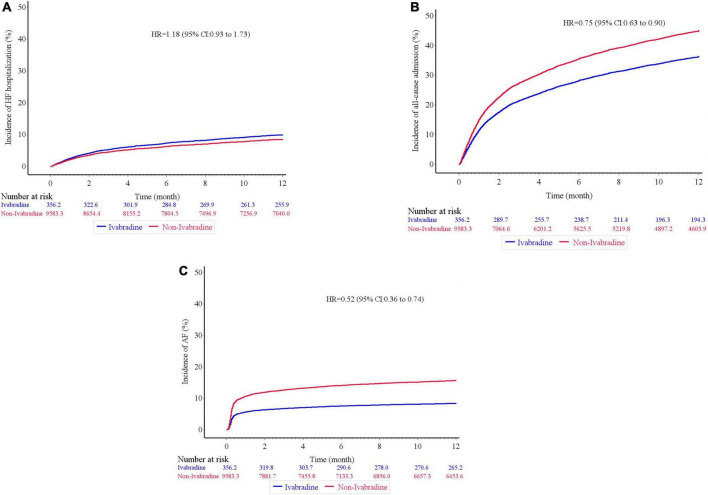
Kaplan–Meier cumulative event curves of clinical outcomes. There was no significant difference in terms of heart failure hospitalization **(A)**, but the ivabradine group had lower incidence rates of all-cause hospitalization **(B)**, and newly developed atrial fibrillation **(C)** compared to the non-ivabradine group after inverse probability of treatment weighting analysis without further adjustments.

**FIGURE 4:**
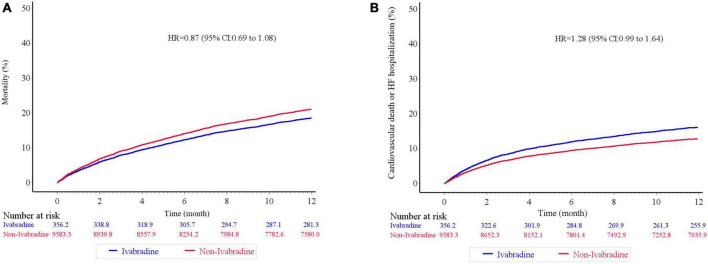
Kaplan–Meier cumulative event curves of clinical outcomes. There was no significant difference in terms of mortality **(A)**, and the composite of cardiovascular death or heart failure hospitalization **(B)** between ivabradine and the non-ivabradine groups after inverse probability of treatment weighting analysis without further adjustments.

There was no significant difference in mortality in the index admission during the index admission between the ivabradine (15.10%; 77/510) and non-ivabradine (15.12%; 1,710/11,311) groups. The clinical outcomes after discharge after IPTW are summarized in Table 2. There was no significant difference in the incidence of HF hospitalization between the two groups at 12 months (8.98% in the ivabradine group and 7.70% in the non-ivabradine group; *P* = 0.375) (Table 2). Kaplan–Meier curves showed that there was no significant difference in the incidence of HF hospitalization between the two groups [hazard ratio (HR) = 1.18; 95% confidence interval (CI), 0.83–1.67; *P* = 0.367] (Figure 3A and Table 3). However, there was a significantly lower incidence of all-cause hospitalization in the ivabradine group compared to the non-ivabradine group at 12 months (35.22% in the ivabradine group vs. 42.60% in the non-ivabradine group; *P* = 0.006) (Table 2). Kaplan–Meier curves showed that the incidence of all-cause hospitalization was lower in the ivabradine group (HR = 0.75; 95% CI, 0.63–0.90; *P* = 0.002) (Figure 3B and Table 3). The incidence of AF was also significantly lower in the ivabradine group at 12 months (5.66% in the ivabradine group vs. 17.97% in the non-ivabradine group; *P* < 0.0001) (Table 2). Kaplan–Meier curves showed that the incidence of AF was lower in the ivabradine group (HR = 0.52; 95% CI, 0.36–0.74; *P* = 0.0004) (Figure 3C and Table 3). Furthermore, there were no significances (HR = 0.87; 95% CI, 0.69–1.08,; *P* = 0.208) in the terms of mortality (Figure 4A and Table 3) as well as the composite of CV death or HF hospitalization (HR = 0.87; 95% CI, 0.69–1.08; *P* = 0.051) (Figure 4B and Table 3) between groups.

**TABLE 2:** The incidence of clinical outcomes at different time points between the ivabradine and non-ivabradine groups after IPTW.

	Group	1 M	*P*-value	3 M	*P*-value	6 M	*P*-value	12 M	*P*-value
HF hospitalization (%)	Ivabradine	2.09	0.991	4.78	0.687	6.47	0.663	8.98	0.375
	Non-ivabradine	2.10		4.33		5.92		7.70	
All-cause hospitalization (%)	Ivabradine	6.98	<0.001	17.82	<0.001	24.24	<0.001	35.22	<0.001
	Non-ivabradine	15.06		26.71		34.24		42.60	
Newly developed AF (%)	Ivabradine	4.72	<0.001	5.03	<0.001	5.66	<0.001	5.66	<0.001
	Non-ivabradine	14.85		16.31		17.21		17.97	
Mortality	Ivabradine	2.10	0.104	6.83	0.169	14.19	0.864	21.21	0.976
	Non-ivabradine	3.86		8.95		13.87		21.14	
CV death or HF hospitalization	Ivabradine	4.35	0.778	11.62	0.559	20.06	0.489	28.17	0.509
	Non-ivabradine	4.22		10.92		18.59		26.58	

AF, atrial fibrillation; CV, cardiovascular; HF, heart failure; IPTW, inverse probability of treatment weighting.

**TABLE 3:** Clinical outcomes between ivabradine and non-ivabradine groups by different adjusted analysis after IPTW.

Outcomes	Crude analysis		Model 1^†^		Model 2^‡^	
	HR (95% CI)	*P*-value	HR (95% CI)	*P*-value	HR (95% CI)	*P*-value
**Primary endpoint**						
Heart failure hospitalization	1.18 (0.83–1.67)	0.367	1.17 (0.82–1.66)	0.382	1.02 (0.38–2.79)	0.964
**Other clinical endpoints**						
All-cause hospitalization	0.75 (0.63–0.90)	0.002	0.75 (0.62–0.89)	0.001	0.95 (0.54–1.68)	0.858
New development of AF	0.52 (0.36–0.74)	0.0004	0.51 (0.35–0.74)	0.0004	0.44 (0.15–1.29)	0.134
Mortality	0.87 (0.69–1.08)	0.208	1.07 (0.86–1.34)	0.547	0.84 (0.54–1.33)	0.460
CV death or HF hospitalization	1.28 (0.99–1.64)	0.051	1.39 (1.09–1.79)	0.009	0.98 (0.63–1.53)	0.927

^†^Model 1 was adjusted by sex, age.

^‡^Model 2 was adjusted by sex, age, history of myocardial infarction, hemoglobin, BNP, heart rate, LVEF, LVEDD, digoxin, loop diuretics, MRA, admission stay. Hazard ratio was presented with non-ivabradine group as reference.

AF, atrial fibrillation; CV, cardiovascular; HR, hazard ratio; IPTW, inverse probability of treatment-weighted.

In further analysis with age-sex adjustments, the incidence rates of the outcomes were similar to those in crude analysis (Table 3; Supplementary Figure 1). However, the differences in HF hospitalization, all-cause hospitalization, and the composite of CV death or HF hospitalization between the groups disappeared after multivariate analysis (Table 3; Supplementary Figure 2).

### Echocardiographic parameters

**FIGURE 5:**
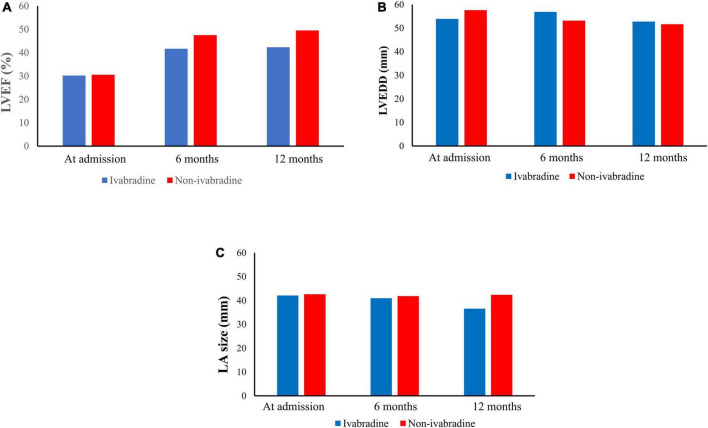
Echocardiographic parameters during the observation period. After discharge, there were 168 patients received echocardiogram in ivabradine group while 1,648 patients received echocardiogram in non-ivabradine group in our observation period. In echocardiographic result, it showed that improvements in left ventricular ejection fraction (LVEF) **(A)** were seen in both groups. However, there were no significant differences in LVEF **(A)**, left ventricular end-diastolic diameter **(B)**, and left atrium size **(C)** after inverse probability of treatment weighting without further adjustments.

There were trends of increasing LVEF through the observation period in both groups, with an average LVEF of 42.33 ± 9.59% in the ivabradine group and 49.52 ± 16.54% in the non-ivabradine group at 12 months. However, the difference did not reach significance (*P* = 0.460) (Figure 5). Furthermore, the LVEDD was comparable between the two groups at 12 months (52.78 ± 11.60 vs. 51.65 ± 14.14 mm; *P* = 0.137) (Figure 5). LA size was significantly smaller in the ivabradine group at 12 months compared to the non-ivabradine group (36.53 ± 3.28 vs. 42.34 ± 8.14 mm; *P* = 0.037) (Figure 5).

After discharge, improvements in left ventricular ejection fraction (LVEF) (Figure 5A) were seen in both groups. However, there were no significant differences in LVEF (Figure 5A), left ventricular end-diastolic diameter (Figure 5B), and left atrium size (Figure 5C) after IPTW without further adjustments.

### Sensitivity analysis

We use multivariable analysis in the population before IPTW as a sensitivity analysis. After adjusting for baseline characteristics that were significantly different between the ivabradine and non-ivabradine groups, there was no difference in the risk of HF hospitalization between the two groups (HR = 1.04; 95% CI, 0.55–1.95) (Supplementary Table 2). In addition, the risk of all-cause hospitalization (HR = 1.03; 95% CI, 0.72–1.46), mortality (HR = 0.77; 95% CI, 0.38–1.55), and the composite of CV death or HF hospitalization (HR = 1.01; 95% CI, 0.50–2.03) were comparable between the two groups (Supplementary Table 2). However, the risk of AF was lower in the ivabradine group than in the non-ivabradine group (HR = 0.44; 95% CI, 0.21–0.91) (Supplementary Table 2).

## Discussion

In this study, the patients who received ivabradine had better HR control than those who did not receive ivabradine during the index admission and at 12 months after discharge. However, there were no significant differences in HF hospitalization, all-cause hospitalization, mortality, the incidence of CV death or HF hospitalization, and LV systolic function between the ivabradine and non-ivabradine groups after discharge. In addition, the incidence of AF was not higher in the ivabradine group than that in the non-ivabradine group.

In our study, HR at admission was significantly higher in the ivabradine group compared to the non-ivabradine group, and the baseline condition in the ivabradine group appeared to be worse than that in the non-ivabradine group based on the higher prevalence of medications commonly used to relieve symptoms associated with acute decompensated HF, such as loop diuretics and digoxin. In addition, the patients in the ivabradine group were younger than those in non-ivabradine group before matching. We supposed that one reason caused such phenomenon—the physicians were willing to prescribe ivabradine in patients with more severity of HF and higher heart rate, particularly young patients, because ivabradine does not impair cardiac contractility and does not affect hemodynamics (11, 12). However, there was no difference in mortality in the index admission and 12-month mortality between the ivabradine and non-ivabradine groups although a higher HR at admission was independently associated with worse outcomes in patients admitted for acute HF (6). Taken those together, these findings suggest the possible benefits of initiating ivabradine in patients with acute decompensated HF.

Heart rate after discharge is also an important issue in HF patients and an important target when treating patients with acute compensated HF. Several studies have demonstrated the safety and efficacy of lowering HR with the early administration of ivabradine in acute HF patients (11, 13, 26). Mentz et al. conducted a randomized, open-label trial to assess the impact of initiating ivabradine prior to discharge in acute HF patients in the background of not reducing β-blocker therapy. The results showed that there was no significant benefit or harm in terms of mortality, HF hospitalization and all-cause hospitalization in short-term follow-up (27). In another small clinical trial, Hidalgo et al. also showed that ivabradine had better HR control, but there were no statistical differences in clinical outcomes such as HF hospitalization or mortality (5, 28). Consistent with these studies, we also found that ivabradine had potentially better HR control than regular management. In terms of HF hospitalization, all-cause hospitalization, and mortality, our results are also similar to the previous studies. There are several possible explanations for these findings. First, the difference in HR was small and the sample size was also small. According to a previous meta-analysis and retrospective cohort study, HR is strongly associated with mortality and recurrent HF hospitalization (29, 30). In our study, although HR was lower in the ivabradine group than that in the non-ivabradine group after discharge, the change in HR combined with the small sample size may not be large enough to show clinical differences. Second, the observation duration may not be long enough to achieve significance. Several previous studies reported that a higher HR at discharge was correlated with a poor prognosis, and that there was no significant interaction between β-blockers and clinical outcomes in short term observation period (less than on year) when the target HR was achieved (31, 32). In our study, HR was significantly lower in the ivabradine group 3 months after discharge compared with the non-ivabradine group, but no significant difference among clinical outcomes was noted in this short observation period. In addition, subsequent analysis in Hidalgo’s study showed the potential benefit of early treatment with ivabradine in long-term outcomes, but it was not evident in short-term outcomes (5, 28).

Several studies have shown that AF is significantly associated with increased mortality in HF patients compared with sinus rhythm (33–35). In addition, the presence of AF was reported to lead to a more severe NYHA class in HF patients in a previous multicenter study (36). Another prospective study further reported that the new onset of AF in HF patients was associated with clinical and hemodynamic deterioration and a worse prognosis (37). According to a prospective observational study (38), the combined administration of ivabradine and β-blockers during the perioperative period was associated with a lower incidence of AF in patients undergoing elective coronary artery bypass graft in short-term follow-up compared with either regular medication alone. In our study, although the incidence of AF was lower in the ivabradine group than in the non-ivabradine group according to crude analysis after IPTW, the benefit became insignificantly after further multivariate analysis. Compared to the results of the SHIFT study, which concluded that ivabradine increased the risk of AF (10), our study showed that the initiation of ivabradine in the acute stage of HF at least did not increase the risk of AF. However, the definition of AF was according to any records in CGMH medical database, but asymptomatic AF may be ignored or missed in our retrospective cohort study. Therefore, as only *de novo* HF patients were included in our study, this may suggest that ivabradine can be considered to be initiated in the early stage of HF.

There were several limitations to this retrospective cohort study. First, the baseline characteristics of the study groups could not be fully matched despite our best efforts, which may have led to selection bias. However, sensitivity analysis confirmed our outcomes. Further prospective studies may be warranted. Second, HR is an important parameter in ivabradine studies. However, we could not guarantee consistent measurement quality at all visits, and variations in HR may not reflect the effect of HR-control agents. Third, clinical presentations, functional assessments, such as 6 min walking test, and some biomarkers for HF, such as NT-pro-B-type natriuretic peptide, were not regularly measured during follow-up. Therefore, the objective severity and functional status of HF could not be assessed. Fourth, although echocardiographic result showed LA size seemly be reduced after treating with ivabradine, not all patients have echocardiographic examination in the follow-up period. In addition, inter and intra-observer variability cannot be avoided in our retrospective observation study. Therefore, further prospective study should be warranted to confirm the finding. Fifth, the study population was from our medical centers and we cannot trace the medical records in other hospitals, with the exception of mortality. However, the follow-up rate was around 60% at the 12-month follow-up period as shown in Supplementary Table 3. Therefore, the population may not be sufficient to draw a solid conclusion and a prospective cohort study should be warranted.

## Conclusion

Ivabradine had better HR control compared to regular pharmacological management, and there were no significant differences in in-hospital mortality and HF/all-cause hospitalization, mortality between the ivabradine group and non-ivabradine group in 12 months after discharge. Furthermore, ivabradine did not contribute to the risk of AF in the acute stage of HF. Therefore, initiating ivabradine during hospitalization for acute HF could be an option for patients with acute decompensated HF.

## Data availability statement

The original contributions presented in this study are included in the article/Supplementary material, further inquiries can be directed to the corresponding author.

## Ethics statement

The studies involving human participants were reviewed and approved by Institutional Review Board of Chang Gung Memorial Hospital (IRB: 201900572B0C602). Written informed consent for participation was not required for this study in accordance with the national legislation and the institutional requirements.

## Author contributions

T-YY and M-ST designed the study, interpreted the data, and wrote the manuscript. J-YJ contributed to conceptualization and acquisition and interpretation of data. J-JC and C-MC contributed to analysis, interpretation of data, and discussion. M-SL and H-MC contributed to analysis of data and reviewed the manuscript. Y-SL contributed to supervision and critical revision of the manuscript for important intellectual content. All authors contributed to the article and approved the submitted version.
