# Supplementary material for: Early administration of ivabradine in patients admitted for acute decompensated heart failure
Source: Front Cardiovasc Med. 2022 Nov 29;9:1036418. doi: 10.3389/fcvm.2022.1036418 (PMC9744812; doi:10.3389/fcvm.2022.1036418)
Supplement: Supplementary file 1 [file Data_Sheet_1.docx]

**Supplemental Table 1.** Changes in HR in the patients who were admitted for heart failure with or without ivabradine administration during the index hospitalization

| Outcomes | Ivabradine group  (*n* = 433) | Non-ivabradine group  (*n* =9601) | *P*-value |
| --- | --- | --- | --- |
| In-hospital HR (bpm) |  |  |  |
| At admission | 97.43 ± 17.46 | 87.23 ± 20.46 | <0.0001 |
| At discharge | 80.59 ± 11.78 | 80.05 ± 15.12 | 0.443 |
| Change in HR* | -16.83 ± 19.99 | -7.19 ± 20.02 | <0.0001 |
| HR after discharge (bpm) |  |  |  |
| 1 month | 77.55 ± 13.27 | 82.59 ± 18.90 | 0.133 |
| 3 months | 74.40 ± 9.92 | 82.23 ± 17.48 | 0.0005 |
| 6 months | 73.14 ± 8.54 | 82.76 ± 17.46 | 0.0005 |
| 12 months | 74.14 ± 8.53 | 81.23 ± 16.79 | 0.079 |

Abbreviations: bpm, beats per minute; HR, heart rate

Data are presented as mean ± standard deviation

* change in HR was defined as subtracting HR at admission from HR at discharge.

**Supplemental Table 2.** Clinical outcomes between the ivabradine and non-ivabradine groups by different adjust analysis before IPTW.

|  | **Crude analysis** | | **Model 1**† | | **Model 2**‡ | | |
| --- | --- | --- | --- | --- | --- | --- | --- |
| **Outcomes** | HR (95% CI) | *P*-value | HR (95% CI) | *P* -value | | HR (95% CI) | *P* -value |
| **Primary endpoint** |  |  |  |  |  | |  |
| Heart failure admission | 1.27 (0.93-1.73) | 0.134 | 1.31 (0.96-1.78) | 0.095 | 1.04 (0.55-1.95) | | 0.905 |
| **Other clinical endpoints** |  |  |  |  |  | |  |
| All-cause admission | 0.89 (0.76-1.04) | 0.132 | 0.95 (0.82-1.11) | 0.515 | 1.03 (0.72-1.46) | | 0.887 |
| New development of AF | 0.61 (0.45-0.84) | 0.002 | 0.68 (0.50-0.93) | 0.015 | 0.44 (0.21-0.91) | | 0.027 |
| Mortality | 1.00(0.79-1.26) | 1.00 | 0.99(0.79-1.26) | 0.974 | 0.77(0.38-1.55) | | 0.459 |
| CV death or HF hospitalization | 0.99(0.73-1.35) | 0.973 | 0.98(0.73-1.34) | 0.933 | 1.01(0.50-2.03) | | 0.976 |
|  |  |  |  |  |  | |  |

†Model 1 was adjusted by sex, age:

‡Model 2 was adjusted by sex, age, history of myocardial infarction, hemoglobin, BNP, heart rate, LVEF, LVEDD, digoxin, loop diuretics, MRA, admission stay.

Hazard ratios were presented with the non-ivabradine group as reference.

Abbreviations: AF, atrial fibrillation; HR, hazard ratio; IPTW, inverse probability of treatment weighting.

**Supplemental table 3: follow-up rate in different stage of follow-up period**

|  | **Ivabradine group (n=433)** | | |  | **Non-ivabradine group (n=9601)** | | | | |
| --- | --- | --- | --- | --- | --- | --- | --- | --- | --- |
|  | Survival patients (n) | Follow-up patients (n) | Follow-up ratio (%) | | |  | Survival patients (n) | Follow-up patients (n) | Follow-up ratio (%) |
| **3 Months** | 417 | 351 | 84.17% | | |  | 9238 | 6720 | 72.74% |
| **6 months** | 400 | 283 | 70.75% | | |  | 8751 | 5640 | 64.45% |
| **9 months** | 375 | 247 | 65.87% | | |  | 8279 | 5107 | 61.69% |
| **12 months** | 352 | 200 | 56.82% | | |  | 7581 | 4721 | 62.27% |


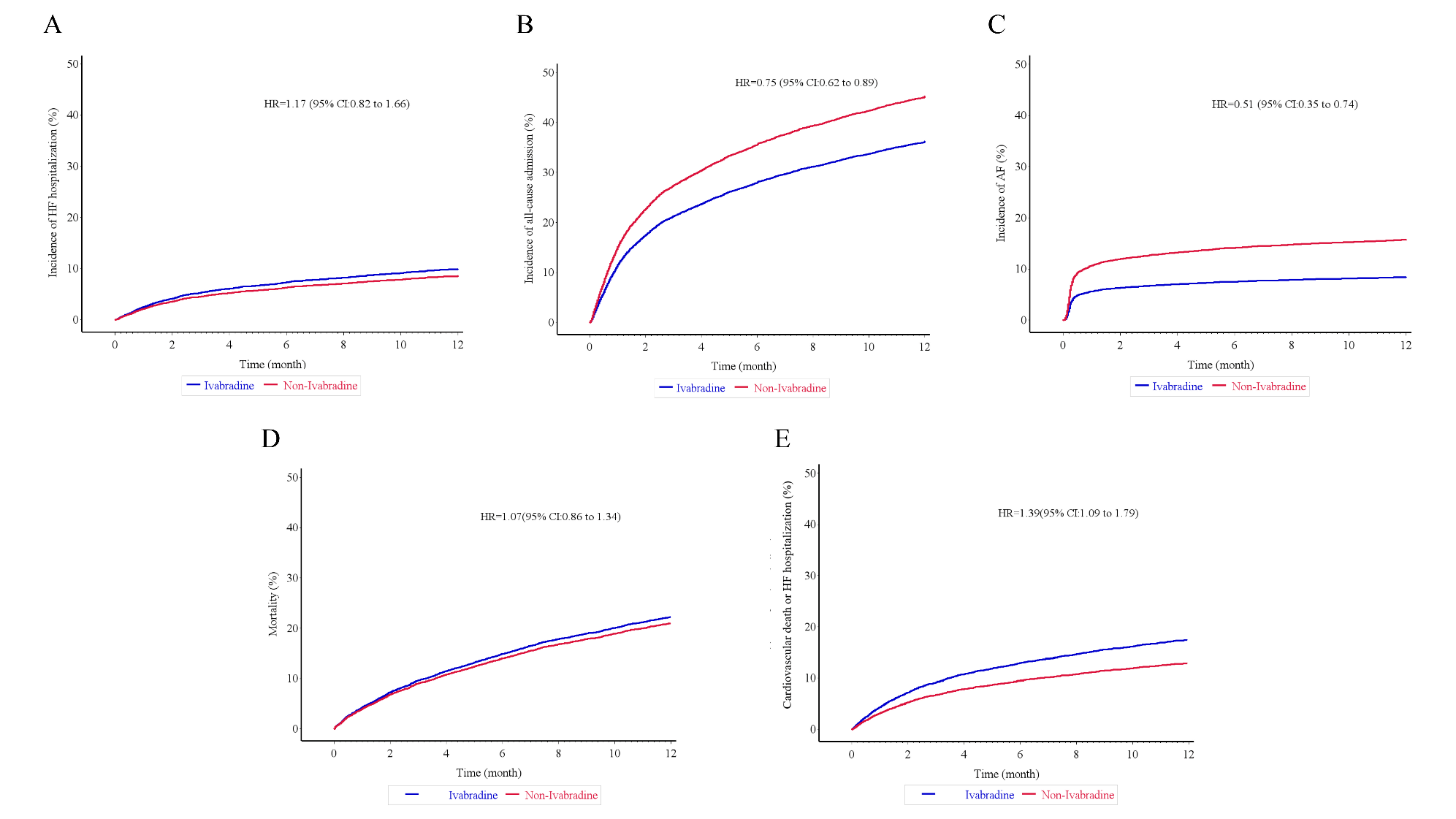
**Supplemental figure 1:** Survival curves of clinical outcomes after inverse probability of treatment weighting analysis with age and sex adjustments. There was no significant difference in terms of heart failure hospitalization **(A)** and mortality**(D)**, but the ivabradine group had lower incidence rates of all-cause hospitalization **(B)** and newly developed atrial fibrillation**(C),** but mild higher incidence rate of the composite of CV death or HF hospitalization**(E)** compared to the non-ivabradine group after adjusting age and sex.


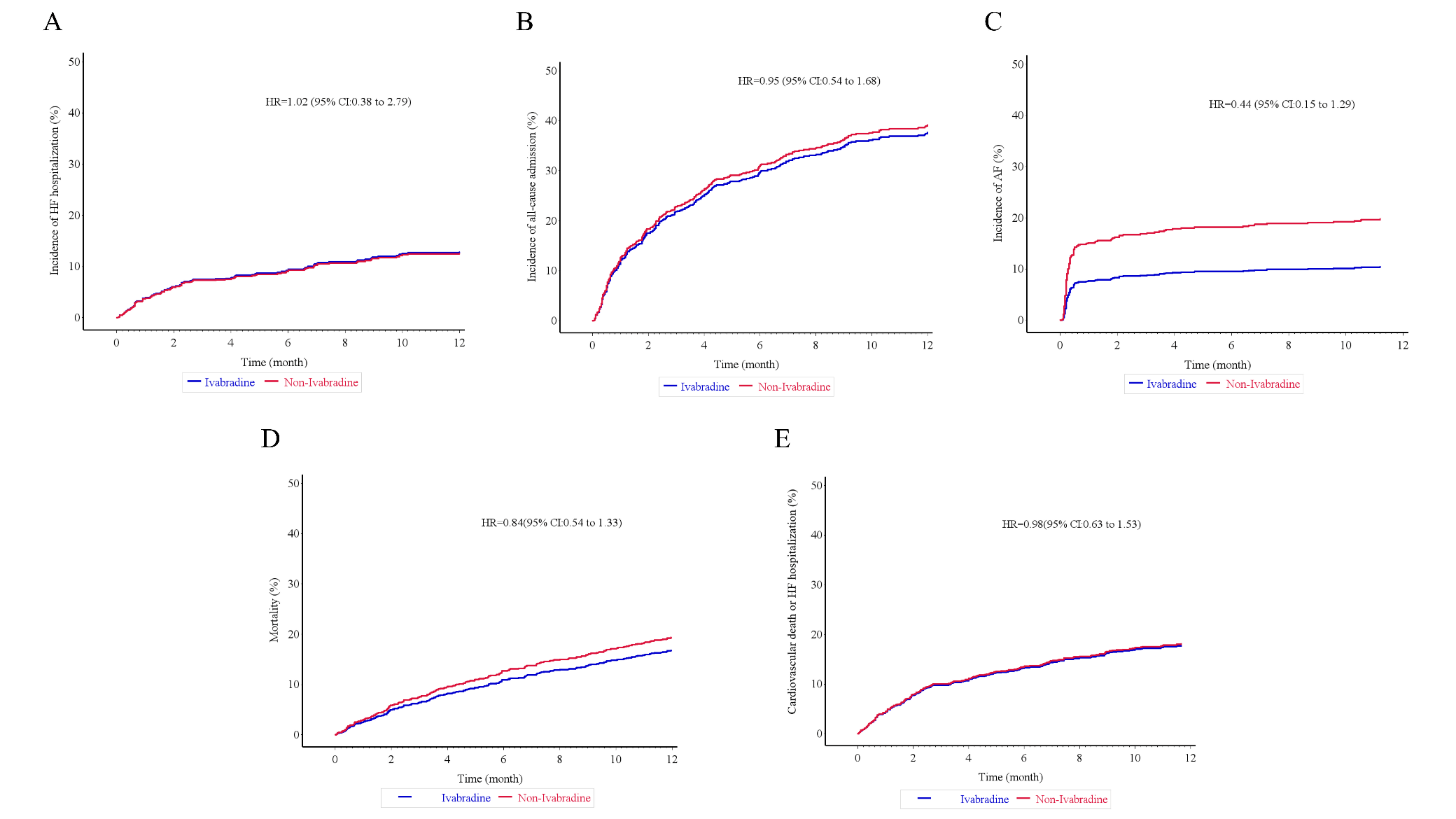
**Supplemental figure 2:** Survival curves of clinical outcomes after inverse probability of treatment weighting analysis with multi-variant adjustments. There was no significant difference in terms of heart failure hospitalization **(A),** all-cause hospitalization **(B)**, newly developed atrial fibrillation**(C),** mortality**(D)** and the composite of CV death or HF hospitalization**(E)** compared to the non-ivabradine group after adjusting multi-variances.
